# Isolated suprasellar involvement in tuberculosis: findings on magnetic resonance imaging

**DOI:** 10.1590/0100-3984.2017.0091

**Published:** 2019

**Authors:** Bernardo Carvalho Muniz, Bruno Niemeyer de Freitas Ribeiro, Nina Ventura, Emerson Leandro Gasparetto, Edson Marchiori

**Affiliations:** 1 Instituto Estadual do Cérebro Paulo Niemeyer - Departamento de Radiologia, Rio de Janeiro, RJ, Brazil.; 2 Universidade Federal do Rio de Janeiro (UFRJ), Rio de Janeiro, RJ, Brazil.

**Figure 1:**
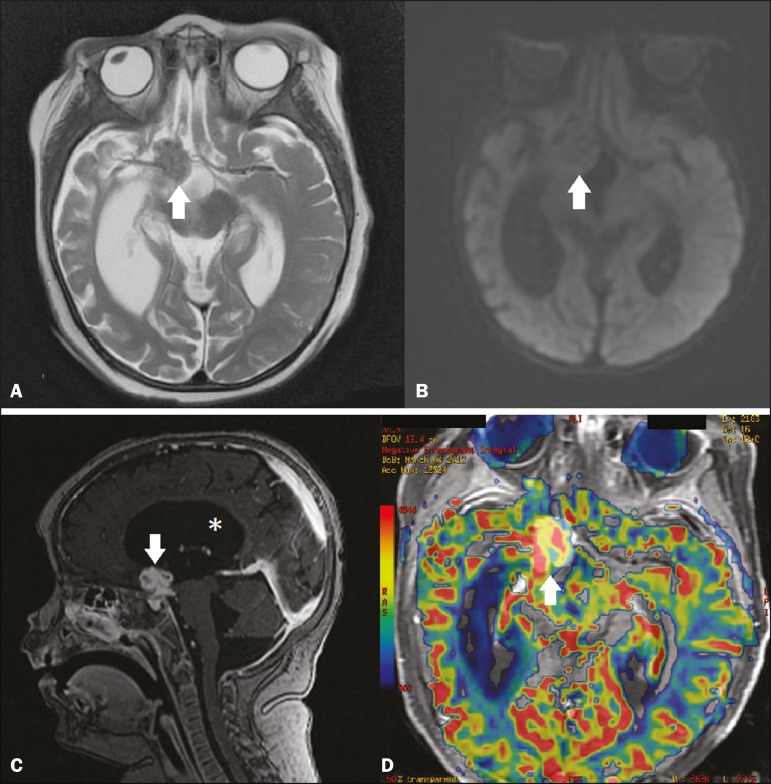
MRI. **A:** Axial T2-weighted sequence, showing a suprasellar lesion with a hypointense signal (arrow) and reduced volume in the right temporal lobe. **B:** Axial diffusion- weighted sequence, showing no restricted diffusion in the lesion (arrow). **C:** Contrast-enhanced sagittal T1-weighted sequence, showing intense contrast enhancement of the lesion, showing some areas with annular uptake (arrow). Note the dilatation of the lateral ventricle (asterisk). **D:** Axial perfusion MRI superimposed on a contrast-enhanced T1-weighted sequence, showing an increase in cerebral blood volume.

Dear Editor,

A two-year-old female patient presented with a one-month history of diffuse headache, lethargy, and a decline in her general health status. Magnetic resonance imaging (MRI) of the skull showed a suprasellar, lobulated, heterogeneous, expansile lesion, with a signal that was predominantly isointense in T1-weighted sequences and hypointense in T2-weighted sequences, with no restricted diffusion and with intense contrast enhancement, with or without areas of annular uptake, as well as significant ectasia of the lateral ventricles, probably due to obstruction of the third ventricle (Figure 1). Computed tomography of the thorax and abdomen showed no alterations. A histopathological study showed a granulomatous chronic inflammatory process with caseous necrosis, and acid-fast bacilli were identified, confirming the diagnosis of suprasellar tuberculosis.

Recent studies in the radiology literature of Brazil have emphasized the importance of imaging examinations for improving central nervous system diagnoses^(^1^-^4^)^. Tuberculosis is an infectious disease caused by *Mycobacterium tuberculosis*, which is still common in low- and middle-income countries like Brazil. The lungs are the main organs affected, followed by the pleurae, lymph nodes, and skeletal system. The central nervous system is affected in 0.15-5.0% of cases, the main manifestation in such cases being meningitis, which occurs in 95% of cases^(^5^,^^6^^)^. Sellar/juxtasellar involvement is even rarer, typically the result of hematogenous spread from a primary source, usually the lungs^(^5^)^. Clinically, it can manifest as visual field defects, hypopituitarism, and central diabetes insipidus^(^7^)^.

On MRI, most suprasellar tuberculomas show a signal that is isointense or hypointense in T1-weighted sequences and hyperintense in T2-weighted sequences; however, there are reports of lesions with a hypointense signal in T2-weighted sequences, which are explained by variations in the degree of hydration of the lesion^(^5^-^8^)^. In addition, the degree of cellularity and the content of these lesions allow findings of either absence or presence of restricted diffusion in diffusion-weighted sequences. Uptake is common after the intravenous administration of contrast medium, sometimes assuming an annular aspect^(^5^-^8^)^. An increase in cerebral blood volume can be seen on perfusion MRI, with a gradual reduction in that volume after pharmacological treatment^(^9^)^.

The diagnosis of suprasellar tuberculoma is made by identifying acid-fast bacilli in a biopsy sample of the lesion; however, in endemic areas, the diagnosis can be made solely on the basis of histopathological findings typical of the disease, including a granulomatous inflammatory process and caseous necrosis^(^5^,^^8^^)^. The differential diagnosis is broad; however, when such findings are seen in a very young individual and in the suprasellar space, the main differential diagnoses are craniopharyngiomas, astrocytomas, germinomas, Langerhans cell histiocytosis, and vasculitis accompanied by myocardial infarctions^(^8^)^.

Suprasellar tuberculoma is treated with a specific tuberculosis treatment regimen, consisting of two months of rifampin, isoniazid, pyrazinamide, and ethambutol, followed by seven months of rifampin and isoniazid accompanied by corticosteroids^(^10^)^. Decompressive surgery may be required in cases of hydrocephalus or compression of vital structures, such as the optic chiasm^(^5^,^^7^^)^.

In conclusion, although rare, a diagnosis of tuberculosis should be considered in suprasellar lesions, especially when there is annular enhancement on MRI, in areas endemic for the disease.
